# The relationship between ego depletion and work alienation in Chinese nurses: A network analysis

**DOI:** 10.3389/fpsyg.2022.915959

**Published:** 2022-07-22

**Authors:** Yi Cui, Tianqi Yang, Hui Gao, Lei Ren, Na Liu, Xufeng Liu, Yinling Zhang

**Affiliations:** ^1^Department of Nursing, Air Force Medical University, Xi’an, China; ^2^Department of Military Medical Psychology, Air Force Medical University, Xi’an, China; ^3^Xi’an International Medical Center Hospital, Xi’an, China

**Keywords:** nurse, ego depletion, work alienation, network analysis, cross-sectional survey

## Abstract

**Objectives:**

To investigate the network structure of ego depletion and work alienation in Chinese nurses and identify bridge items to provide suggestions to reduce ego depletion and work alienation.

**Methods:**

A total of 353 nurses from three hospitals were enrolled in our cross-sectional study by convenience sampling from June to October 2021 in China. They completed an online survey, which included the Sociodemographic Questionnaire, Nurses’ Work Alienation Questionnaire, and Self-Regulating Fatigue Scale (SRF-S). The R packages qgraph, networktools, and bootnet were used to estimate the network model and calculate the indices.

**Results:**

The correlation between ego depletion and work alienation was mainly positive. The correlation between “Sometimes I do not know what to do with the work instructions from my superiors” and “I have difficulties remembering things” was the strongest among the cross-community correlations (*r* = 0.14). The bridge strength centrality indices of “Sometimes I do not know what to do with the work instructions from my superiors,” “I always feel like a loser” and “I have difficulties remembering things” were the highest (*z score* = 3.15, 2.83, 1.43). The correlation stability coefficient of the centrality index was larger than 0.25.

**Conclusion:**

Nurses’ ego depletion and work alienation are correlated. “Sometimes I do not know what to do with the work instructions from my superiors,” “I always feel like a loser” and “I have difficulties remembering things” act as bridges between ego depletion and work alienation communities, and should be the focus of nurses’ psychological tests. Our study provides potential targets for interventions to reduce work alienation from the perspective of ego depletion.

## Introduction

With the continuous spread of the COVID-19 pandemic, the shortage of global health human resources is becoming increasingly obvious, among which the demand for nursing personnel is particularly high (Sriharan et al., 2021). Fighting against the novel coronavirus on the front line, nurses have shown a high sense of responsibility and dedication to people’s health. However, according to the 2020 World Nursing Report released by the World Health Organization, a comprehensive analysis of the data of 191 countries showed that nursing departments were seriously understaffed (Ma et al., 2021). There were many challenges for nurses during the COVID-19 outbreak; for example, nurses were coming into close contact with patients, which put them at very high risk of infection. It was necessary to wear personal protective equipment, which increased the difficulty of providing nursing care by requiring more time and energy. In addition, because patients were in a state of isolation, the scope of nursing functions increased along with work pressure (Labrague and de Los Santos, 2021). Under these highly intense conditions, humanistic care for nurses is lacking, leaving nurses to cope with their negative emotions (Mo et al., 2020). Over time, an increasing number of nurses want to leave the current work environment, and there is a sense of work alienation (Raso et al., 2021). Therefore, it is of great significance to identify work alienation as soon as possible and to ensure the vitality of nursing teams and reduce the loss of nurses.

Work alienation is a very important social psychological problem at present and refers to the subjective negative psychological state that results from the separation of employees from their work, which is caused by the failure of the work environment to meet employees’ material and spiritual needs (Amzulescu and Butucescu, 2021). Nurses easily feel alienated from their work because of the enormous workload, high intensity, high risk and low reward and the pressure to achieve promotions and produce scientific research publications (Zhang et al., 2021). When nurses are in a state of work alienation, their work autonomy, participation in decision-making, and nursing quality decrease, while their negative emotions and turnover intentions increase. Therefore, it is very important to identify and prevent work alienation in the early stage (Amarat et al., 2019; Bacaksiz et al., 2020). Work alienation is a complex concept composed of a variety of negative psychological states, which mainly fall into three dimensions and multiple items: sense of helplessness, friendlessness, and meaninglessness (Banai et al., 2004). However, in the literature, most studies have regarded work alienation as a whole and explored the relationship between its total score and external factors such as work stress and social support (Aeschbacher and Addor, 2018). The dimension most easily affected by other factors and the most important part of work alienation have not been identified, and work alienation has seldom been considered from the aspect of nurses’ psychological resources. Therefore, attention should be given to exploring the relationship between the specific items of work alienation and other influencing factors and whether the consumption of nurses’ psychological energy leads to work alienation.

Ego depletion is also called self-regulating fatigue (SRF). According to the theory of limited self-control resources, individuals consume limited self-control resources when they perform self-regulating behaviors, which may lead to a series of complex cognitive, emotional, and behavioral problems (Baumeister et al., 1998). In the face of a heavy workload, patients’ diversified health service needs, complex management, and interpersonal relationships, nurses must adjust and adapt constantly (Lorente et al., 2021). Their internal psychological resources are consumed constantly, resulting in different degrees and expressions of ego depletion, further resulting in emotional exhaustion, a fear of workdays, an indifference to patients, a reduced sense of personal achievement, and feelings of worthlessness (Molina-Praena et al., 2018). Ultimately, it is easy for work alienation to affect the quality of nursing (Sasso et al., 2019). Some studies have found that employees with a serious degree of ego depletion have a higher sense of work alienation (Zhao and Liu, 2019, 2020). However, there is no research on the relationship between nurses’ ego depletion and work alienation in China or abroad, and the specific correlation mechanism between their structures is not clear.

Network analysis is an innovative method that can be used for mathematical analysis and an intuitive display of the relationship between complex variables; in particular, it plays a unique and important role in exploring the finer-grained correlation path of two related variables (Galderisi et al., 2018; Wei et al., 2021). It is data-driven rather than depending on a priori hypotheses of causality between variables (Ren et al., 2021). In the psychological network, nodes represent psychological variables, such as emotions, symptoms, and attitudes, while edges represent relationships between variables. In recent years, network analysis has received extensive attention and has been applied to various fields of psychology, such as developmental psychology (Goh et al., 2020), psychiatry (Vanzhula et al., 2021), and health psychology (Verkuilen et al., 2021). Consequently, network analysis was suitable for the two variables in our study, and it has the following scientific importance: (1) Network theory provides an alternative method to conceptualize psychological constructs, regarding psychological constructs as interactive systems, as their components interact with each other and actively participate in the emergence of the construct, in contrast to the passive indicators of the construct (Peng et al., 2020). As mentioned above, these two variables are complex psychological constructs. Thus, it is reasonable to see work alienation and ego depletion as two interacting systems consisting of different components. (2) In terms of theoretical contributions, network theory makes up for the defects based on the latent variable model, such as the overall level, and attaches importance to the interaction between components level. (3) At the same time, compared with simple related methods, the network model can also provide a centrality indicator for each node to quantify its importance in the whole network. In this case, accurately describing these interactions and locating the most important nodes in the network may help nursing managers develop more targeted intervention strategies.

At present, most of the research on work alienation and ego depletion is based on the latent variable model, ignoring the complex interaction between the items of the two variables (Wu et al., 2021). Therefore, this paper intended to use the advantages of network analysis to provide new perspectives for the study of the relationship between work alienation and ego depletion in more detail and to make more targeted clinical interventions. Based on the above, our study had three aims. First, we wanted to explore the potential pathways between different components of nurses’ work alienation and ego depletion. Second, the bridge centrality index was used to investigate which component of work alienation has the strongest connection with ego depletion and which component of ego depletion has the strongest connection with work alienation. Finally, we attempted to improve the understanding of the complex relationships between work alienation and ego depletion and provide some theoretical support for interventions to reduce them.

## Materials and methods

### Subjects and procedures

From June to October 2021, 353 nurses from three grade 3A hospitals in Xi’an, Shaanxi Province, China, were selected to participate in the cross-sectional descriptive survey by the convenience sampling method. Nurses who were registered nurses, engaged in clinical nursing work (including clinical nursing and nursing management), and had been working for at least1 year were included. Advanced nurses, trainee nurses, and nurses who were unable to participate in the survey because they were on leave or out of work were excluded. The nurses were all informed and voluntarily participated in this study.

According to Kendall (1975) sample estimation method, the sample size should be 5–10 times that of the independent variable. The Sociodemographic Questionnaire of our study contained 11 items, the Nurses’ Work Alienation Questionnaire contained 3 dimensions, and the Self-Regulating Fatigue Scale (SRF-S) contained 3 dimensions. A total of 17 variables needed to be analyzed, so the sample size was 85–170. Given the possibility of non-response, incomplete questionnaires and lost samples, the sample size increased by 20%, yielding a final minimum sample size of 102 participants.

Nurses completed the questionnaire anonymously by scanning QuickMark or opening the link to the questionnaire powered by www.wjx.cn. The quality control methods included explaining the purpose and significance of the survey at the beginning of the questionnaire and using unified instructions; we also designated a person in charge for each hospital and trained him or her to first ensure a full understanding of the purpose, significance, and standards of the survey and then to mobilize, guide and supervise the nurses in their hospitals to complete the questionnaire. The survey could only be submitted after all the questions had been answered. The questionnaires were collected and checked by the researchers, and those that did not meet the standards were excluded. In this study, a total of 361 questionnaires were received, of which 353 were validated for an effective rate of 97.78%.

### Measures

#### Sociodemographic questionnaire

The questionnaire was designed by the researchers according to the purpose of the study, and included items on sex, age, working years, department, education level, employment type, marital status, child status, professional title, and monthly income.

#### Nurses’ work alienation questionnaire

The questionnaire, which was compiled by Chinese scholar Ren (2012), has 12 items and 3 dimensions (sense of helplessness, friendlessness, and meaninglessness). It has been adopted by many scholars, showing good reliability and validity (Chen et al., 2017). Each item uses a 5-point Likert scale scoring system with a score of 1–5, ranging from “strongly disagree” to “strongly agree.” The higher the total score is, the higher the sense of work alienation. In this study, the Cronbach’s α coefficient of the total questionnaire was 0.883, and the Cronbach’s α coefficient of the three dimensions was 0.740, 0.811, and 0.868, respectively.

#### Self-regulating fatigue scale

The scale was developed by Australian scholar Nes et al. (2013) and adapted to Chinese by Wang et al. (2015). Sixteen items are used to reflect individual-specific depletion, including cognition (6 items), emotion (5 items), and behavior (5 items). Each item uses a 5-point Likert scale, with a score of 1–5 indicating “strongly disagree” to “strongly agree.” The higher the total score is, the more serious the individual’s ego depletion. The scale can distinguish ego depletion from self-control and physical fatigue well. In this study, Cronbach’s α = 0.842 for the total scale and Cronbach’s α = 0.288, 0.739 and 0.776 for the three dimensions.

### Statistical analysis

First, the data were analyzed in the SPSS 26.0 package program. Descriptive statistics were conducted to describe sociodemographic characteristics, ego depletion and work alienation. In addition, we performed the comparison of ego depletion and work alienation among different sociodemographic subgroups using independent *t*-test or one-way ANOVA, with *P* < 0.05 as the significant deference evaluation standard.

Then, the R package qgraph (Epskamp et al., 2012) was used to establish a network model based on Spearman correlation. To obtain a more stable and comprehensive network, the graphical least absolute shrinkage and selection operator (LASSO) algorithm was used to conduct the regularization of the Gaussian graphical model, in which all edges were shrunk, and the edge with small partial correlation was set to zero (Friedman et al., 2008; Karthik et al., 2012; Epskamp and Fried, 2018). The tuning parameter was set to 0.5 to balance the sensitivity and specificity. Extended Bayesian Information Criterion (EBIC) model selection was used for regularization estimation of partial correlation networks. After that, we used the R package networktools (Jones et al., 2019) to estimate bridge strength and to identify the nodes bridging the two communities of ego depletion and work alienation.

We examined the robustness of the network by running the R package bootnet (Epskamp et al., 2018). We performed a bootstrapped significance test of the centrality indices of different nodes (2,000 bootstrap samples and α = 0.05). We also conducted case-dropping bootstrapping to evaluate the stability of the centrality indices, whose results can also be presented with the correlation stability coefficient (CS -coefficient), which quantifies the proportion of data that can be dropped to retain, with 95% certainty, a correlation of at least 0.7 with the original centrality coefficients. Ideally, this coefficient should be above 0.5 and must be at least above 0.25. Finally, we applied non-parametric bootstrapping to estimate the 95% confidence intervals (CIs) to evaluate the accuracy of edge weights (2,000 bootstrap samples): In 95% of the cases, such a CI will contain the true value of the parameter.

### Ethical considerations

The research was approved by the Ethics Committee of the First Affiliated Hospital of Air Force Medical University (KY20212203-F-1). Before conducting the survey, we explained the purpose to the participants and asked for their verbal consent; questionnaires were completed anonymously. During the investigation, participants could withdraw at any time.

## Results

### Sociodemographic characteristics and comparison of ego depletion and work alienation

The average age of the participants was 29.93 years (*SD* = 6.25), and the average number of working years was 8.05 years (*SD* = 7.36). For ego depletion and work alienation, we observed statistically significant differences only in the department item (*F* = 2.746, 2.807, *P* = 0.013, 0.011), and there were no significant differences in other sociodemographic characteristics. The sociodemographic characteristics and the comparison of nurses’ ego depletion and work alienation are presented in Table 1.

**TABLE 1:** Sociodemographic characteristics of the participants and the comparison of ego depletion and work alienation (*N* = 353).

Variable	Category	*n* (%)		Ego depletion (M ± *SD*)	Work alienation (M ± *SD*)
**Sex**					
	Male	22 (6.2%)		40.41 ± 8.91	31.14 ± 9.20
	Female	331 (93.8%)		42.00 ± 8.64	30.89 ± 8.22
			*t*	–0.979	0.136
			*P*	0.328	0.892
**Age**					
	18 years∼	194 (55.0%)		41.58 ± 8.74	30.96 ± 8.20
	30 years∼	134 (38.0%)		42.53 ± 8.75	31.43 ± 8.30
	40 years∼	21 (6.0%)		40.71 ± 8.13	27.48 ± 8.40
	50 years∼	4 (1.0%)		41.50 ± 2.65	28.50 ± 9.18
			*F*	0.458	1.501
			*P*	0.712	0.214
**Department**					
	Internal medicine	105 (29.8%)		43.76 ± 8.11	32.18 ± 8.51
	Surgery	66 (18.7%)		42.71 ± 8.52	32.98 ± 8.30
	Obstetrics-gynecology	33 (9.3%)		39.18 ± 8.08	27.39 ± 7.90
	Pediatrics	53 (15.0%)		40.83 ± 8.01	29.66 ± 6.43
	Operating room	16 (4.5%)		38.69 ± 8.18	29.00 ± 8.16
	Intensive care unit	39 (11.1%)		43.13 ± 10.44	31.13 ± 8.48
	Others	41 (11.6%)		39.37 ± 8.73	29.24 ± 8.80
			*F*	2.746	2.807
			*P*	0.013*	0.011*
**Working years**					
	1 year∼	250 (70.8%)		41.86 ± 8.87	31.07 ± 8.01
	11 years∼	89 (25.2%)		42.19 ± 8.49	30.87 ± 9.09
	21 years∼	10 (2.8%)		39.90 ± 6.28	28.00 ± 7.30
	31 years∼	4 (1.2%)		41.50 ± 2.65	28.50 ± 9.18
			*F*	0.214	0.556
			*P*	0.887	0.644
**Education level**					
	High school diploma	84 (23.8%)		42.68 ± 8.42	31.35 ± 7.94
	Bachelor’s degree	261 (73.9%)		41.62 ± 8.80	30.79 ± 8.41
	Master’s degree	8 (2.3%)		42.38 ± 6.30	30.13 ± 8.06
			*F*	0.489	0.181
			*P*	0.613	0.835
**Marital status**					
	Married	196 (55.5%)		42.49 ± 8.97	30.98 ± 8.44
	Unmarried	153 (43.3%)		41.10 ± 8.30	30.81 ± 8.15
	Divorced	2 (0.6%)		42.00 ± 5.66	28.00 ± 8.49
	Widowed	2 (0.6%)		43.00 ± 5.66	33.50 ± 0.71
			*F*	0.750	0.159
			*P*	0.523	0.924
**Employment type**					
	Permanent	43 (12.2%)		41.49 ± 9.38	30.28 ± 8.40
	Contract	310 (87.8%)		41.94 ± 8.56	30.99 ± 8.27
			*t*	–0.322	–0.528
			*P*	0.748	0.598
**Child status**					
	Does not have a child	197 (55.8%)		41.14 ± 8.33	30.97 ± 8.13
	Has a child	113 (32.0%)		42.45 ± 9.14	30.55 ± 8.58
	Has two children or more	43 (12.2%)		43.81 ± 8.61	31.51 ± 8.25
			*F*	2.046	0.226
			*P*	0.131	0.798
**Professional title**					
	Junior	94 (26.6%)		41.28 ± 7.96	30.35 ± 7.74
	Middle	255 (72.3%)		42.08 ± 8.96	31.16 ± 8.49
	Senior	4 (1.1%)		44.00 ± 3.46	27.50 ± 5.92
			*F*	0.414	0.670
			*P*	0.662	0.512
**Monthly income (CYN)**					
	≤4,000	97 (27.5%)		41.76 ± 8.62	30.31 ± 7.52
	4,001–6,000	140 (39.7%)		42.59 ± 8.98	31.77 ± 8.23
	6,001–8,000	61 (17.3%)		42.74 ± 8.68	30.93 ± 8.13
	≥8,001	55 (15.5%)		39.38 ± 7.55	29.71 ± 9.68
			*F*	2.060	1.063
			*P*	0.105	0.365

*P < 0.05.

### Statistical results of the mean and standard deviation of the items in ego depletion and work alienation networks

According to the statistical description results, the means and standard deviations of all variables in the network are presented in Table 2.

**TABLE 2:** Statistical results of the mean and standard deviation of items in the ego depletion and work alienation networks (*N* = 353).

Item	*M* ($\bar{x}$)	*SD* (s)
**Ego depletion**	41.89	8.66
E1. I feel full of energy.	2.80	0.96
E2. It is easy for me to set goals.	2.70	0.87
E3. I find it difficult to exercise as much as I should.	3.30	1.10
E4. I have urges to hit, throw, break, or smash things.	2.24	1.15
E5. I have no trouble making decisions.	2.48	0.90
E6. I experience repeated unpleasant thoughts.	2.52	1.03
E7. I get upset easily.	2.54	1.05
E8. I try not to talk or think about things that bother me.	2.72	1.03
E9. I handle stress well.	2.67	0.82
E10. I experience uncontrollable temper outbursts.	2.46	0.99
E11. I can easily keep up with my friendships and relationships.	3.33	0.96
E12. I cry easily.	2.50	1.06
E13. I have difficulties remembering things.	2.65	1.01
E14. I find it easy to stick to a healthy diet.	3.00	1.05
E15. I feel moody.	2.19	0.98
E16. I have urges to beat, injure, or harm someone.	1.78	0.86
**Nurses’ Work Alienation**	30.90	8.27
W1. Sometimes I do not know what to do with the work instructions from my superiors.	2.91	1.05
W2. Promotion is a slim hope for me.	3.22	1.06
W3. At work, sometimes I have to do things I do not want to do.	3.59	1.07
W4. Promotion is possible only if I have good interpersonal relationships within the unit.	3.05	1.13
W5. I cannot make bosom friends at work at all.	2.46	1.11
W6. I do not get along well with my colleagues.	1.81	0.81
W7. Arguing with colleagues about work issues will affect our relationship.	2.51	1.09
W8. When there is something wrong with my work, I often do not get sincere help from my colleagues.	2.10	0.96
W9. I do not like my job. It does not realize my value.	2.33	1.02
W10. My work does not match my interests.	2.60	1.06
W11. I do the same job every day. It does not make any sense.	2.22	1.06
W12. I always feel like a loser.	2.12	1.06

### Network model of ego depletion and work alienation items

**FIGURE 1:**
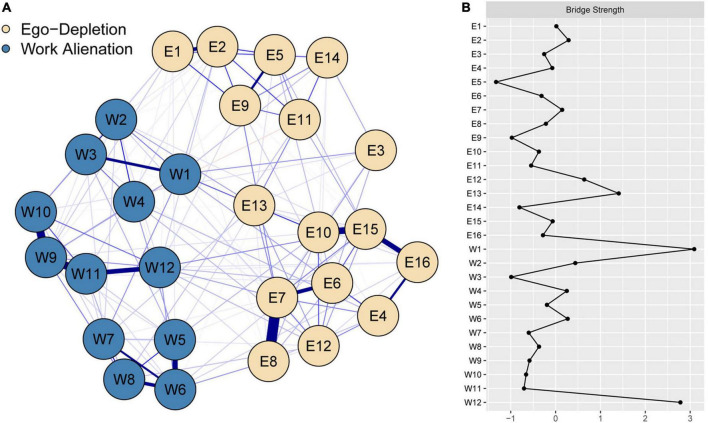
Psychological network of ego depletion and work alienation items. **(A)** The blue line represents positive partial correlation while the red line represents negative partial correlation. The thicker the line and the more saturated the color, the larger the partial correlation coefficient. **(B)** Bridge Strength (*z score*) of Ego depletion and Work Alienation Items. Network. E1, I feel full of energy; E2, It is easy for me to set goals; E3, I find it difficult to exercise as much as I should; E4, I have urges to hit, throw, break, or smash things; E5, I have no trouble making decisions; E6, I experience repeated unpleasant thoughts; E7, I get upset easily; E8, I try not to talk or think about things that bother me; E9, I handle stress well; E10, I experience uncontrollable temper outbursts; E11, I can easily keep up with my friendships and relationships; E12, I cry easily; E13, I have difficulties remembering things; E14, I find it easy to stick to a healthy diet; E15, I feel moody; E16, I have urges to beat, injure, or harm someone; W1, Sometimes I do not know what to do with the work instructions from my superiors; W2, Promotion is a slim hope for me; W3, At work, sometimes I have to do things I do not want to do; W4, Promotion is possible only if I have a good interpersonal relationship in the unit; W5, I cannot make bosom friends at work at all; W6, I do not get along well with my colleagues; W7, Arguing with colleagues about work issues will affect our relationship; W8 = When there is something wrong with my work, I often do not get sincere help from my colleagues; W9, I do not like my job. It does not realize my value; W10, My work doesn’t match my interests; W11, I do the same job every day. It does not make any sense; W12, I always feel like a loser.

The psychological network of the ego depletion and work alienation items is shown in Figure 1A (see Supplementary Table 1 for more information on the correlation matrix).

In the ego depletion community, there were two clusters whose internal nodes are relatively closely correlated and an isolated node. Cluster 1 consisted of the nodes of E1 (I feel full of energy), E2 (It is easy for me to set goals), E5 (I have no trouble making decisions), E9 (I handle stress well), E11 (I can easily keep up with my friendships and relationships) and E14 (I find it easy to stick to a healthy diet), among which the non-zero correlations between nodes were all positive. Cluster 2 consisted of the nodes of E4 (I have urges to hit, throw, break, or smash things), E6 (I experience repeated unpleasant thoughts), E7 (I get upset easily), E8 (I try not to talk or think about things that bother me), E10 (I experience uncontrollable temper outbursts), E12 (I cry easily), E13 (I have difficulties remembering things), E15 (I feel moody), and E16 (I have urges to beat, injure, or harm someone), among which the non-zero correlations were all positive. In addition, E3 (I find it difficult to exercise as much as I should) was relatively isolated. The items belonging to the three dimensions (cognition, emotion, and behavior) of the scale did not correspond to those from the two clusters and the isolated node. The following node pairs had a strong correlation in this community: E7–E8 (*r* = 0.39), E10–E15 (*r* = 0.31), E15–E16 (*r* = 0.28), E6–E7 (*r* = 0.26), and E1–E2 (*r* = 0.25).

The work alienation community contained three clusters whose internal nodes were relatively closely correlated. Cluster 3 consisted of the nodes of W1 (Sometimes I do not know what to do with the work instructions from my superiors), W2 (Promotion is a slim hope for me), W3 (At work, sometimes I have to do things I do not want to do), and W4 (Promotion is possible only if I have good interpersonal relationships within the unit). Similarly, Cluster 4 consisted of the nodes of W5 (I cannot make bosom friends at work at all), W6 (I do not get along well with my colleagues), W7 (Arguing with colleagues about work issues will affect our relationship), and W8 (When there is something wrong with my work, I often do not get sincere help from my colleagues) and Cluster 5 consisted of the nodes of W9 (I do not like my job. It does not realize my value), W10 (My work does not match my interests), W11 (I do the same job every day. It does not make any sense), and W12 (I always feel like a loser). The items contained in the three clusters completely corresponded to the items contained in the three factors of the Nurses’ Work Alienation Questionnaire, namely, helplessness, friendlessness, and meaninglessness. The following node pairs had a strong correlation in this community: W9–W10 (*r* = 0.34), W9–W11 (*r* = 0.33), W5–W6 (*r* = 0.28), W11–W12 (*r* = 0.27), and W6–W8 (*r* = 0.25).

There were also correlations between nodes from different communities that were mainly positive. The following node pairs were the relatively strong pairs in the cross-community correlations: W1–E13 (*r* = 0.14), W12–E15 (*r* = 0.10), W1–E3 (*r* = 0.09), and W6–E8 (*r* = 0.09). Generally, nodes within communities were more closely connected than those across communities. A total of 229 (60.6%) of the 378 possible edges had zero values in the entire network, while 140 (72.9%) of the 192 possible edges had zero values in the network of cross-community nodes.

### Estimation of centrality indices

We calculated the bridge strength (*z* score) of the ego depletion and work alienation item network. As suggested in Figure 1B, the bridge strength indices of nodes W1 (Sometimes I do not know what to do with the work instructions from my superiors), W12 (I always feel like a loser), and E13 (I have difficulties remembering things) were more than one standard deviation larger than the average (see Supplementary Table 2 for the raw scores and *z* scores of the centrality indices).

### Significance test of centrality indices

**FIGURE 2:**
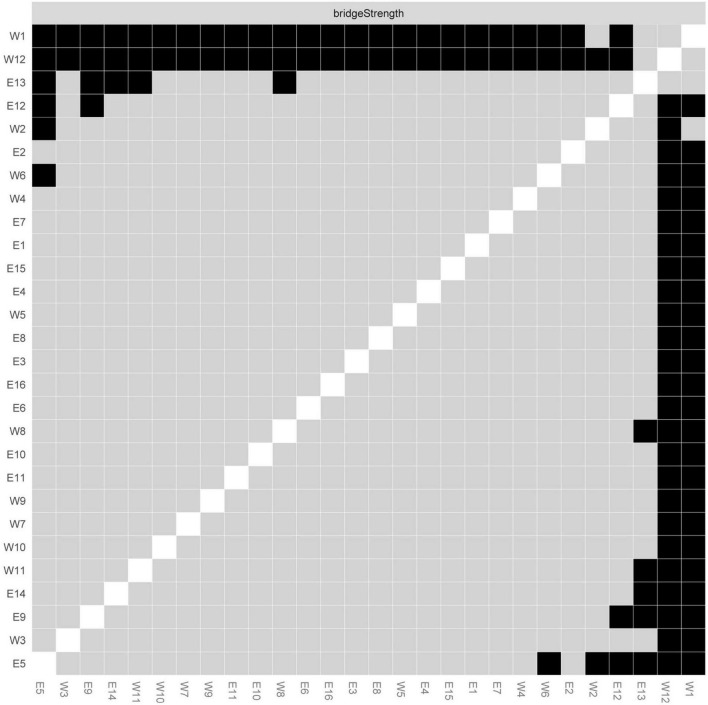
Significance test of bridge strength centrality. The black box indicates that the bridge strength indices of the two corresponding nodes have a significant difference (*P* < 0.05). The gray box indicates no significant difference (*P* > 0.05).

We tested whether there was a significant difference in the bridge strength indices of different nodes, as shown in Figure 2. The bridge strength indices of the nodes of W1 (Sometimes I do not know what to do with the work instructions from my superiors), W12 (I always feel like a loser), and E13 (I have difficulties remembering things) were significantly different from those of some nodes (*P* < 0.05).

### Evaluation of the accuracy of the edge weights and the stability of the centrality indices

**FIGURE 3:**
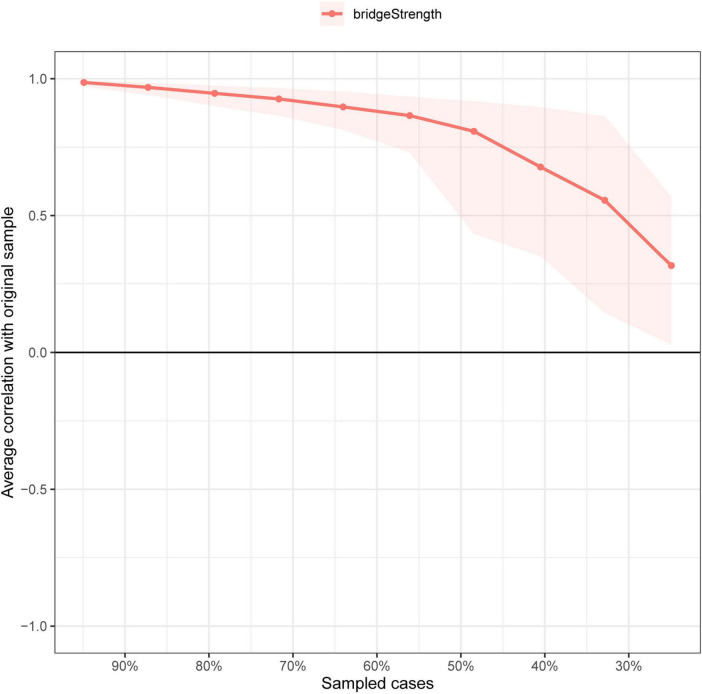
Evaluation of the stability of strength centrality. The red bar represents the average correlation between centrality indices in the full sample and subsample with the red area depicting the 2.5th quantile to the 97.5th quantile.

**FIGURE 4:**
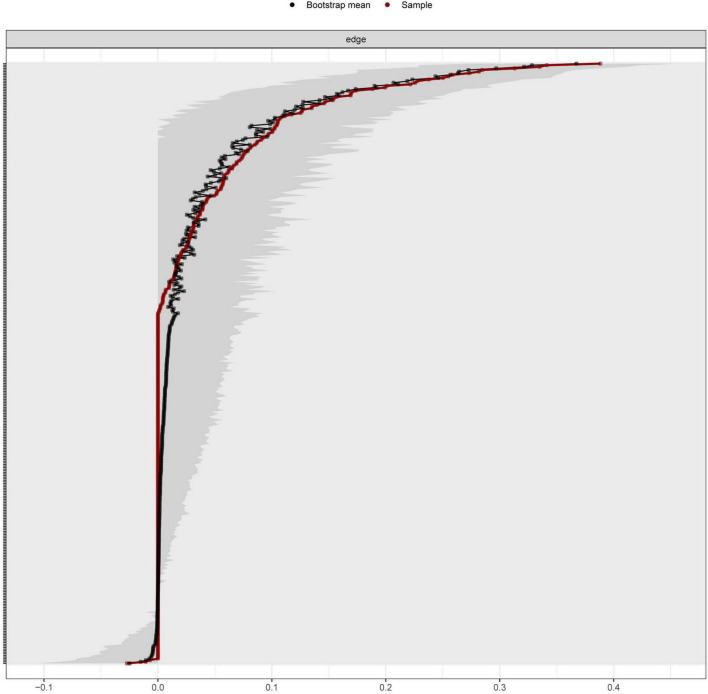
The accuracy of edge weights. The red line indicates the sample values and the gray area indicates the bootstrapped CIs. The y-axis labels have been removed to avoid cluttering.

We conducted case-dropping bootstrapping to evaluate the stability of the bridge strength, as shown in Figure 3. With the dropping of the sample cases, the average correlation with the bridge strength of original sample decreased slightly. The CS - coefficients of bridge strength were also calculated. The result showed that the CS - coefficient of bridge strength was 0.439, which was greater than 0.25, and hence, the stability was acceptable. The accuracy of the edge weights was also estimated, as shown in Figure 4.

## Discussion

In the present study, we used the network analysis method to explore the potential relationship between nurses’ ego depletion and work alienation, and we obtained some meaningful findings. In the community of ego depletion, “I get upset easily” was most strongly associated with “I try not to talk or think about things that bother me.” “I feel moody” and “I experience uncontrollable temper outbursts” had the second strongest association. We found that being upset and moody were negative emotions, which made nurses more prone to negative behaviors such as avoidance, tantrums, and causing injury. This is consistent with the results of a study with nursing students by Rutherford et al. (2020). The regulation of nurses’ negative emotions is very important to their work behavior and quality of nursing (Sun et al., 2021). It is suggested that nursing managers should not only pay attention to nurses’ work performance but also identify nurses’ negative emotions in a timely manner, prevent impulsive behaviors and help nurses use emotional regulation strategies to master psychological balance and deal with work rationally (Wang et al., 2021). Especially during the COVID-19 outbreak, the number of patients increased sharply, and nurses were at risk of infection at any time during their clinical work (Chen et al., 2022). We should keep a watchful eye not only on patients’ conditions but also on nurses’ physical and mental states.

In the community of work alienation, “My work does not match my interests” and “I do not like my job. It does not realize my value” had the strongest correlation. “I do the same job every day. It does not make any sense” and “I do not like my job. It does not realize my value” had the second strongest correlation, which was similar to the findings in a study with ICU nurses by Hao et al. (2020). Work interest affects nurses’ sense of work value and stimulates the meaning of work, thus affecting nurses’ work alienation. It is suggested that in the process of training nurses, we should pay attention to improving nurses’ professional identities, encouraging their interest and enthusiasm in nursing work, and supporting them in fully reflecting their inner values (Poorchangizi et al., 2019). It is also necessary to improve the social awareness and status of the nursing industry so that nurses can truly feel the significance of their clinical nursing work and realize their self-worth (Rasheed et al., 2019).

Items from different communities of ego depletion and work alienation also had a correlation with each other, and the non-zero correlation was mainly positive. “I have difficulties remembering things” in the cognitive dimension and “Sometimes I do not know what to do with the work instructions from my superiors” in the helplessness dimension had the strongest correlation. This suggests that these two items, which connect ego depletion and work alienation, are probably the most important channels through which the two interact with each other. This was in line with the results of Bedyńska et al. (2018), which revealed that a decline in working memory is likely to lead to a sense of helplessness. Clinical nursing work is often accompanied by trivial and busy tasks, and nurses must negotiate relationships with patients, doctors, and their colleagues at the same time (Broetje et al., 2020). In addition, they have to face all kinds of assessments, continuing education requirements and so on, resulting in serious depletion of psychological resources. As a result, they may overlook some details in their work, be unable to reasonably understand instructions from their superiors, and feel an increasing sense of helplessness. Eventually, all these factors might lead to work alienation. Therefore, nursing managers should pay attention to the rational distribution of work tasks, intensity, and difficulty and avoid assigning a large number of tasks to the same nurse at the same time, which would decrease the nurse’s work burden, lessen their ego depletion, and reduce their sense of work alienation (Tomlin et al., 2020). The “I feel moody” and “I always feel like a loser” pair also had a relatively strong correlation. Moody people are usually unable to control their emotions or bring their emotions to work. This practice affects not only their work judgment but also their interpersonal relationships, leading to a sense of meaninglessness (Giménez-Espert et al., 2020). It also suggests that nurse managers should have regular heart-to-heart talks with nurses, be good at identifying nurses’ emotional changes and learn to listen. Nurses should also be encouraged to vent their feelings, appropriately express their dissatisfaction, and look at problems from different perspectives. Only by maintaining a steady emotional state can a person maintain efficient work.

Bridge strength centrality represents the sum of the connection strength between a node and all nodes in other communities (Jones et al., 2019), reflecting the role of nodes in different community connections. Therefore, bridge strength can better reflect the relationship of community connections and thus provide a more effective interference target. This study shows that the bridge strength centrality of three nodes (“I have difficulties remembering things,” “Sometimes I do not know what to do with the work instructions from my superiors” and “I always feel like a loser”) was high. These three nodes act as a bridge between ego depletion and work alienation and provide an important potential target for interventions to reduce ego depletion and work alienation. Therefore, when intervening in nurses’ work alienation from the perspective of ego depletion, starting with the above three items may be more effective than starting with others. For example, nurses can make daily or weekly work plans, arrange each task to prevent forgetfulness, and reduce the increase in ego depletion caused by the need to remember a large number of trivial things, which would ultimately lead to reductions in the sense of work alienation (Yu et al., 2016). Nursing managers should create a reasonable and equitable division of labor, promptly communicate with nurses, and improve nurses’ confidence in accomplishing various tasks. When nurses encounter difficulties, they should be assisted in solving the problems in a timely manner instead of being blamed for them. Additionally, we must encourage nurses to realize the importance and value of their nursing work and improve their sense of professional achievement to reduce ego depletion at work and lessen the sense of work alienation.

## Implications for nursing managers

This study is of great significance to nursing managers. Nursing managers should identify nurses’ work alienation as soon as possible to reduce the turnover rate of nurses, maintain the vitality of nursing teams and improve the quality of nursing care. The lower nurses’ consumption of psychological resources is, the lower their sense of work alienation. From the perspective of important nodes found in the study, nursing managers should focus on the following aspects. On the one hand, nursing managers should pay more attention to nurses’ physical and mental health, encourage them to learn emotional regulation methods, reduce the occurrence of negative emotions, distribute nurses’ work tasks fairly, and reduce work pressure and psychological burden. On the other hand, nursing managers can improve nurses’ sense of professional value through reward mechanisms, oral praise, and making them aware of the importance of nursing work by helping them explore the positive aspects of their job so that they can maintain strong interest and enthusiasm in their nursing work.

## Limitations

Our study provides a specific intervention target for reducing nurses’ ego depletion and work alienation. Additionally, we can provide some theoretical references for reducing the turnover rate of nurses and ensuring the vitality of nursing teams. However, our study also has some limitations. First, the centrality indices used were relatively simple, and only bridge strength centrality was used; centrality indices such as bridge betweenness and closeness betweenness were not used because of poor stability, which is a typical problem of the network analysis method (Forbes et al., 2017). Second, there was no clear quantitative operational definition of the bridge, so we could only determine the bridge in accordance with the requirements of the study. However, results with different definitions may not be comparable. Finally, due to the limited content, scale-based tests may conceal actual psychological problems and may need to be further combined with open interviews to determine the reliability of the results.

## Conclusion

In summary, our study reveals the characteristics of the network structure of nurses’ ego depletion and work alienation based on the perspective of network analysis. This enriches the mechanism of the relationship between nurses’ ego depletion and work alienation. The results provide more targeted theoretical guidance and a scientific basis for psychological counseling and interventions to achieve the goal of reducing nurses’ ego depletion and work alienation. However, all the above conclusions require more confirmatory studies in the future to validate them.

## Data availability statement

The raw data supporting the conclusions of this article will be made available by the authors, without undue reservation.

## Ethics statement

The studies involving human participants were reviewed and approved by the Ethics Committee of the First Affiliated Hospital of Air Force Medical University (KY20212203-F-1). Written informed consent for participation was not required for this study in accordance with the national legislation and the institutional requirements.

## Author contributions

YC and YZ designed the study. YC and TY contributed to the writing of all parts of the manuscript. HG and NL were responsible for collecting the data. TY and LR contributed to the data analysis. YZ and XL reviewed the manuscript and made constructive suggestions. All authors contributed to the article and approved the submitted version.

## Conflict of interest

The authors declare that the research was conducted in the absence of any commercial or financial relationships that could be construed as a potential conflict of interest.

## Publisher’s note

All claims expressed in this article are solely those of the authors and do not necessarily represent those of their affiliated organizations, or those of the publisher, the editors and the reviewers. Any product that may be evaluated in this article, or claim that may be made by its manufacturer, is not guaranteed or endorsed by the publisher.
