# Supplementary material for: The relationship between ego depletion and work alienation in Chinese nurses: A network analysis
Source: Front Psychol. 2022 Jul 22;13:915959. doi: 10.3389/fpsyg.2022.915959 (PMC9355549; doi:10.3389/fpsyg.2022.915959)
Supplement: Supplementary file 1 [file Table_1.docx]

**Table S1.** [Correlation](javascript:;) [Matrix](javascript:;) of Ego depletion and Work Alienation Items

|  | **E1** | **E2** | **E3** | **E4** | **E5** | **E6** | **E7** | **E8** | **E9** | **E10** | **E11** | **E12** | **E13** | **E14** | **E15** | **E16** | **W1** | **W2** | **W3** | **W4** | **W5** | **W6** | **W7** | **W8** | **W9** | **W10** | **W11** | **W12** |
| --- | --- | --- | --- | --- | --- | --- | --- | --- | --- | --- | --- | --- | --- | --- | --- | --- | --- | --- | --- | --- | --- | --- | --- | --- | --- | --- | --- | --- |
| **E1** |  | 0.25 | 0.00 | 0.00 | 0.08 | 0.00 | 0.00 | 0.01 | 0.17 | 0.00 | 0.00 | 0.00 | 0.00 | 0.13 | 0.00 | 0.00 | 0.00 | 0.02 | 0.00 | 0.01 | 0.02 | 0.00 | 0.00 | 0.00 | 0.04 | 0.04 | 0.00 | 0.01 |
| **E2** | 0.25 |  | 0.00 | 0.00 | 0.17 | 0.00 | 0.00 | 0.00 | 0.15 | 0.00 | 0.15 | 0.00 | 0.00 | 0.01 | 0.00 | 0.00 | 0.05 | 0.02 | 0.01 | 0.00 | 0.00 | 0.00 | 0.00 | 0.00 | 0.01 | 0.00 | 0.04 | 0.04 |
| **E3** | 0.00 | 0.00 |  | 0.00 | 0.00 | 0.07 | 0.08 | 0.00 | 0.00 | 0.03 | 0.00 | 0.00 | 0.02 | 0.07 | 0.00 | 0.00 | 0.09 | 0.00 | 0.00 | 0.00 | 0.00 | 0.00 | 0.00 | 0.00 | 0.00 | 0.03 | 0.00 | 0.00 |
| **E4** | 0.00 | 0.00 | 0.00 |  | 0.00 | 0.16 | 0.10 | 0.05 | 0.00 | 0.02 | 0.00 | 0.10 | 0.00 | 0.00 | 0.00 | 0.23 | 0.07 | 0.00 | 0.00 | 0.06 | 0.00 | 0.00 | 0.00 | 0.00 | 0.00 | 0.00 | 0.00 | 0.00 |
| **E5** | 0.08 | 0.17 | 0.00 | 0.00 |  | 0.06 | 0.00 | 0.00 | 0.23 | 0.00 | 0.10 | 0.00 | 0.03 | 0.06 | 0.06 | 0.00 | 0.00 | 0.00 | 0.00 | 0.00 | 0.00 | 0.00 | 0.00 | 0.00 | 0.00 | 0.00 | 0.00 | 0.00 |
| **E6** | 0.00 | 0.00 | 0.07 | 0.16 | 0.06 |  | 0.26 | 0.06 | 0.04 | 0.00 | 0.00 | 0.11 | 0.00 | 0.00 | 0.13 | 0.00 | 0.03 | 0.00 | 0.00 | 0.00 | 0.00 | 0.00 | 0.00 | 0.00 | 0.00 | 0.00 | 0.02 | 0.06 |
| **E7** | 0.00 | 0.00 | 0.08 | 0.10 | 0.00 | 0.26 |  | 0.39 | 0.10 | 0.02 | 0.00 | 0.11 | 0.10 | 0.00 | 0.11 | 0.00 | 0.00 | 0.04 | 0.02 | 0.00 | 0.02 | 0.00 | 0.01 | 0.00 | 0.00 | 0.00 | 0.00 | 0.08 |
| **E8** | 0.01 | 0.00 | 0.00 | 0.05 | 0.00 | 0.06 | 0.39 |  | 0.00 | 0.06 | 0.00 | 0.02 | 0.02 | 0.00 | 0.00 | 0.00 | 0.00 | 0.00 | 0.00 | 0.00 | 0.00 | 0.09 | 0.00 | 0.00 | 0.00 | 0.00 | 0.00 | 0.03 |
| **E9** | 0.17 | 0.15 | 0.00 | 0.00 | 0.23 | 0.04 | 0.10 | 0.00 |  | 0.00 | 0.13 | 0.00 | 0.00 | 0.10 | 0.01 | 0.00 | 0.00 | 0.00 | 0.00 | -0.01 | 0.00 | 0.00 | 0.00 | 0.00 | 0.00 | 0.00 | 0.00 | 0.03 |
| **E10** | 0.00 | 0.00 | 0.03 | 0.02 | 0.00 | 0.00 | 0.02 | 0.06 | 0.00 |  | 0.00 | 0.14 | 0.17 | 0.02 | 0.31 | 0.00 | 0.02 | 0.00 | 0.00 | 0.00 | 0.06 | 0.00 | 0.00 | 0.00 | 0.03 | 0.00 | 0.00 | 0.00 |
| **E11** | 0.00 | 0.15 | 0.00 | 0.00 | 0.10 | 0.00 | 0.00 | 0.00 | 0.13 | 0.00 |  | 0.00 | 0.10 | 0.15 | 0.07 | 0.00 | 0.00 | 0.00 | 0.00 | -0.03 | 0.00 | 0.01 | 0.00 | 0.05 | 0.00 | 0.00 | 0.00 | 0.00 |
| **E12** | 0.00 | 0.00 | 0.00 | 0.10 | 0.00 | 0.11 | 0.11 | 0.02 | 0.00 | 0.14 | 0.00 |  | 0.08 | 0.02 | 0.08 | 0.03 | 0.01 | 0.01 | 0.00 | 0.06 | 0.00 | 0.00 | 0.02 | 0.05 | 0.00 | 0.00 | 0.00 | 0.06 |
| **E13** | 0.00 | 0.00 | 0.02 | 0.00 | 0.03 | 0.00 | 0.10 | 0.02 | 0.00 | 0.17 | 0.10 | 0.08 |  | 0.00 | 0.03 | 0.03 | 0.14 | 0.07 | 0.00 | 0.00 | 0.03 | 0.00 | 0.04 | 0.00 | 0.00 | 0.00 | 0.00 | 0.00 |
| **E14** | 0.13 | 0.01 | 0.07 | 0.00 | 0.06 | 0.00 | 0.00 | 0.00 | 0.10 | 0.02 | 0.15 | 0.02 | 0.00 |  | 0.00 | 0.00 | 0.05 | 0.00 | 0.00 | 0.00 | 0.00 | 0.00 | 0.00 | 0.00 | 0.00 | 0.00 | 0.00 | 0.00 |
| **E15** | 0.00 | 0.00 | 0.00 | 0.00 | 0.06 | 0.13 | 0.11 | 0.00 | 0.01 | 0.31 | 0.07 | 0.08 | 0.03 | 0.00 |  | 0.28 | 0.00 | 0.00 | 0.00 | 0.00 | 0.00 | 0.04 | 0.00 | 0.00 | 0.00 | 0.00 | 0.00 | 0.10 |
| **E16** | 0.00 | 0.00 | 0.00 | 0.23 | 0.00 | 0.00 | 0.00 | 0.00 | 0.00 | 0.00 | 0.00 | 0.03 | 0.03 | 0.00 | 0.28 |  | 0.00 | 0.03 | -0.01 | 0.00 | 0.00 | 0.04 | 0.00 | 0.00 | 0.00 | 0.00 | 0.02 | 0.02 |
| **W1** | 0.00 | 0.05 | 0.09 | 0.07 | 0.00 | 0.03 | 0.00 | 0.00 | 0.00 | 0.02 | 0.00 | 0.01 | 0.14 | 0.05 | 0.00 | 0.00 |  | 0.11 | 0.24 | 0.06 | 0.00 | 0.00 | 0.09 | 0.00 | 0.07 | 0.00 | 0.00 | 0.00 |
| **W2** | 0.02 | 0.02 | 0.00 | 0.00 | 0.00 | 0.00 | 0.04 | 0.00 | 0.00 | 0.00 | 0.00 | 0.01 | 0.07 | 0.00 | 0.00 | 0.03 | 0.11 |  | 0.20 | 0.19 | 0.01 | 0.00 | 0.04 | 0.00 | 0.03 | 0.01 | 0.00 | 0.03 |
| **W3** | 0.00 | 0.01 | 0.00 | 0.00 | 0.00 | 0.00 | 0.02 | 0.00 | 0.00 | 0.00 | 0.00 | 0.00 | 0.00 | 0.00 | 0.00 | -0.01 | 0.24 | 0.20 |  | 0.12 | 0.07 | 0.00 | 0.03 | 0.00 | 0.02 | 0.09 | 0.00 | 0.00 |
| **W4** | 0.01 | 0.00 | 0.00 | 0.06 | 0.00 | 0.00 | 0.00 | 0.00 | -0.01 | 0.00 | -0.03 | 0.06 | 0.00 | 0.00 | 0.00 | 0.00 | 0.06 | 0.19 | 0.12 |  | 0.08 | 0.00 | 0.00 | 0.00 | 0.07 | 0.00 | 0.00 | 0.00 |
| **W5** | 0.02 | 0.00 | 0.00 | 0.00 | 0.00 | 0.00 | 0.02 | 0.00 | 0.00 | 0.06 | 0.00 | 0.00 | 0.03 | 0.00 | 0.00 | 0.00 | 0.00 | 0.01 | 0.07 | 0.08 |  | 0.28 | 0.06 | 0.17 | 0.03 | 0.00 | 0.04 | 0.00 |
| **W6** | 0.00 | 0.00 | 0.00 | 0.00 | 0.00 | 0.00 | 0.00 | 0.09 | 0.00 | 0.00 | 0.01 | 0.00 | 0.00 | 0.00 | 0.04 | 0.04 | 0.00 | 0.00 | 0.00 | 0.00 | 0.28 |  | 0.22 | 0.25 | 0.03 | 0.00 | 0.03 | 0.09 |
| **W7** | 0.00 | 0.00 | 0.00 | 0.00 | 0.00 | 0.00 | 0.01 | 0.00 | 0.00 | 0.00 | 0.00 | 0.02 | 0.04 | 0.00 | 0.00 | 0.00 | 0.09 | 0.04 | 0.03 | 0.00 | 0.06 | 0.22 |  | 0.20 | 0.00 | 0.04 | 0.04 | 0.00 |
| **W8** | 0.00 | 0.00 | 0.00 | 0.00 | 0.00 | 0.00 | 0.00 | 0.00 | 0.00 | 0.00 | 0.05 | 0.05 | 0.00 | 0.00 | 0.00 | 0.00 | 0.00 | 0.00 | 0.00 | 0.00 | 0.17 | 0.25 | 0.20 |  | 0.11 | 0.00 | 0.07 | 0.00 |
| **W9** | 0.04 | 0.01 | 0.00 | 0.00 | 0.00 | 0.00 | 0.00 | 0.00 | 0.00 | 0.03 | 0.00 | 0.00 | 0.00 | 0.00 | 0.00 | 0.00 | 0.07 | 0.03 | 0.02 | 0.07 | 0.03 | 0.03 | 0.00 | 0.11 |  | 0.34 | 0.33 | 0.09 |
| **W10** | 0.04 | 0.00 | 0.03 | 0.00 | 0.00 | 0.00 | 0.00 | 0.00 | 0.00 | 0.00 | 0.00 | 0.00 | 0.00 | 0.00 | 0.00 | 0.00 | 0.00 | 0.01 | 0.09 | 0.00 | 0.00 | 0.00 | 0.04 | 0.00 | 0.34 |  | 0.15 | 0.13 |
| **W11** | 0.00 | 0.04 | 0.00 | 0.00 | 0.00 | 0.02 | 0.00 | 0.00 | 0.00 | 0.00 | 0.00 | 0.00 | 0.00 | 0.00 | 0.00 | 0.02 | 0.00 | 0.00 | 0.00 | 0.00 | 0.04 | 0.03 | 0.04 | 0.07 | 0.33 | 0.15 |  | 0.27 |
| **W12** | 0.01 | 0.04 | 0.00 | 0.00 | 0.00 | 0.06 | 0.08 | 0.03 | 0.03 | 0.00 | 0.00 | 0.06 | 0.00 | 0.00 | 0.10 | 0.02 | 0.00 | 0.03 | 0.00 | 0.00 | 0.00 | 0.09 | 0.00 | 0.00 | 0.09 | 0.13 | 0.27 |  |
