# Supplementary material for: The relationship between ego depletion and work alienation in Chinese nurses: A network analysis
Source: Front Psychol. 2022 Jul 22;13:915959. doi: 10.3389/fpsyg.2022.915959 (PMC9355549; doi:10.3389/fpsyg.2022.915959)
Supplement: Supplementary file 2 [file Table_2.docx]

**Table S2.** Raw Scores and Z-scores of Centrality Indices

| **Item** | **Strength Raw Score** | **Strength Z-score** | **Bridge Strength Raw Score** | **Bridge Strength Z-score** |
| --- | --- | --- | --- | --- |
| E1 | 0.7900377 | -0.46387776 | 0.144480722 | 0.016873553 |
| E2 | 0.9120969 | 0.143954857 | 0.173040686 | 0.29532965 |
| E3 | 0.3846708 | -2.4825312 | 0.116183049 | -0.259025239 |
| E4 | 0.7920664 | -0.453775203 | 0.135029062 | -0.075278956 |
| E5 | 0.7837021 | -0.495427896 | 0.004070945 | -1.352104308 |
| E6 | 0.9906658 | 0.53521369 | 0.110017810 | -0.319135552 |
| E7 | 1.4290628 | 2.718351021 | 0.158065891 | 0.149327261 |
| E8 | 0.7327905 | -0.748958405 | 0.12017688 | -0.220085883 |
| E9 | 0.9714723 | 0.439633551 | 0.040829005 | -0.993717795 |
| E10 | 0.8655439 | -0.087870616 | 0.103844974 | -0.379319935 |
| E11 | 0.7890054 | -0.469018426 | 0.085806065 | -0.555197054 |
| E12 | 0.8918877 | 0.043316717 | 0.209431581 | 0.65013635 |
| E13 | 0.8729309 | -0.0510847 | 0.289801212 | 1.433730255 |
| E14 | 0.5956671 | -1.431807999 | 0.058536193 | -0.821074914 |
| E15 | 1.2186796 | 1.670680947 | 0.135869449 | -0.067085287 |
| E16 | 0.6881215 | -0.971401901 | 0.113317583 | -0.286963176 |
| W1 | 1.0321373 | 0.741734218 | 0.465581915 | 3.147570245 |
| W2 | 0.7936550 | -0.445864264 | 0.188585164 | 0.446886377 |
| W3 | 0.8025226 | -0.401705229 | 0.039293088 | -1.008692794 |
| W4 | 0.6834485 | -0.994672591 | 0.168833518 | 0.254310285 |
| W5 | 0.8661575 | -0.084815 | 0.122548081 | -0.196966968 |
| W6 | 1.0744768 | 0.952577228 | 0.170881319 | 0.27427609 |
| W7 | 0.7977432 | -0.425505772 | 0.080183009 | -0.610021151 |
| W8 | 0.9004132 | 0.085772156 | 0.104141094 | -0.376432802 |
| W9 | 1.2115173 | 1.635013995 | 0.081974588 | -0.592553478 |
| W10 | 0.8215544 | -0.306930326 | 0.073953769 | -0.670755466 |
| W11 | 0.9973254 | 0.568377287 | 0.069176674 | -0.717331548 |
| W12 | 1.0399463 | 0.780621618 | 0.433348888 | 2.833302241 |
